# Disruption of host-associated and benthic microbiota affects reproductive output and settlement of a habitat-forming macroalga

**DOI:** 10.1098/rspb.2025.0729

**Published:** 2025-06-25

**Authors:** Alexander Harry McGrath, Peter D. Steinberg, Suhelen Egan, Staffan Kjelleberg, Ezequiel M. Marzinelli

**Affiliations:** ^1^ School of Life and Environmental Sciences, The University of Sydney, Sydney, New South Wales 2006, Australia; ^2^ Sydney Institute of Marine Science, Mosman, New South Wales 2088, Australia; ^3^ University of New South Wales, Sydney, New South Wales 2033, Australia; ^4^ Singapore Centre for Environmental Life Sciences Engineering, Nanyang Technological University, Singapore

**Keywords:** experimental microbial ecology, microbiome variation, life history, holobiont, host–microbe interactions

## Abstract

The reproduction and establishment of habitat-forming species are key processes affecting their persistence and associated biodiversity. In marine systems, microbial communities associated with habitat-forming macroalgae can influence various aspects of host performance; however, the role of these microorganisms in influencing macroalgal reproduction and settlement is poorly understood. Using a dominant habitat-forming macroalga on Australian rocky shores, *Hormosira banksii,* we manipulated host- and benthic-associated microbiota to determine the relative importance of microorganisms to reproductive output (number of viable eggs released) and settlement (settlement and morphogenesis of algal zygotes). Disruption of the host microbiota using antibiotics decreased reproductive output after 2 weeks, with the effect dependent on the type of antibiotic used. Disruption of host- and benthic-associated microbiota, in combination, caused a significant decrease in settlement of *H. banksii* zygotes, with the combined disruption having the greatest impact on settlement success. Our results demonstrate the importance of host-associated microbiota in macroalgal reproduction and an interactive effect of host- and benthic-associated microbiota on settlement—a key ecological process with important implications for host fitness and potentially ecosystem persistence.

## 1. Introduction

A central challenge in ecology is to understand the factors affecting dynamics of natural populations throughout their life cycle [1,2]. This is especially relevant for habitat-forming organisms whose survival and persistence can underpin broader biodiversity and ecosystem function [3,4]. In the marine environment, the persistence and success of habitat-forming organisms such as corals, sponges and macroalgae are often dependent on their reproductive output and early life-history stages: settlement, morphogenesis and subsequent survival of their early developmental stages [5–7]. Indeed, an understanding of recruitment of marine organisms, i.e. the arrival and success of early life-history stages into the adult habitat or ‘supply-side ecology’ has been a central feature of our understanding of marine ecology and evolution for many years [8].

The life cycles of many marine habitat-forming organisms are complex and often biphasic, with a planktonic propagule phase (e.g. algal zygotes or spores, invertebrate larvae) followed by a sessile or low-motility post-settlement benthic stage [9–11]. Settlement involves the transition between the planktonic propagule and the benthic phase [12], which includes propagule attachment to the substratum and morphogenesis [9,13], i.e. cell differentiation and development of the settling propagule [14]. Settlement can be induced by environmental cues such as light intensity and attenuation [15–17], surface chemistry and wettability [18], acoustics [19] and surface topography [20–22] that may indicate suitable habitat [23–25]. Furthermore, cues from conspecifics or other macroorganisms can either induce (e.g. [26]) or inhibit settlement [27,28].

Microbial biofilms can also play an important role in settlement and the early life stages of marine macroorganisms (reviewed in [29]). Settlement can be affected by the density of biofilm bacteria [30–33], bacterial quorum-sensing molecules [34–36], age of the biofilm [37–39], the specific taxa present [21,40] and other properties of microbial biofilms (e.g. [9,41]). While there is strong evidence that microbial biofilms induce settlement in invertebrates ranging from sponges to echinoderms [29,42], we know much less about the role microorganisms play in the production, settlement and morphogenesis of algal propagules [34,35]. There is some evidence that reproduction or early life-history phases of several algal species are affected by surface-dwelling microorganisms [43–48]. Algal oogonia and antheridia development can at least in part be controlled by exudates released from bacterial consortia [49–51]. Furthermore, evidence from a number of algal species or genera such as *Ulva*, *Ectocarpus, Chondrus crispus* and *Fucus* has shown that microbial communities are fundamentally important for the normal development and function of the algal host [52–55]. However, in general, we still have limited knowledge about the impact of microbial communities on early life-history stages of macroalgae such as propagule release, settlement, attachment, morphogenesis and which microbial taxa are important for these effects.

One reason for focusing on the role host-associated microbiota has in reproduction and early life-history stages of holobionts (i.e. host plus its microbiota [56]) is that these processes or stages are key to the fitness of the host. Performance of holobionts in response to manipulation of their microbiota is often measured via physiological or molecular parameters, such as photosynthetic yield [57–60], nutrient content [61,62] or stress marker genes [63,64]. Ultimately, in assessing performance we want to know if the microbiota affects the fitness of the holobiont, i.e. reproduction and/or survival. While fitness measures typically rely on multigenerational data which is difficult to collect for slow-growing, long-lived species, proxies have been used to accurately predict lifetime fitness, including survival [65] and reproductive output and development [26]. Here, to expand on previously published work for this species (see [58]), we focus on reproductive output and settlement of propagules in order to move more closely to measures of actual host fitness.

We examined the effect of host-associated and benthic microbial communities on the reproduction and early life-history stages of the dioecious (separate sexes) alga *Hormosira banksii* (*Hormosira* hereafter, see electronic supplementary material, figure S1 for *Hormosira*’s life cycle). *Hormosira* is an ideal system to test ideas about the importance of host-associated microbial communities as it is the primary intertidal habitat-forming macroalgae within southeast Australia. Understanding the factors mediating its persistence and survival is fundamental to our understanding of how these ecosystems will persist within a changing climate. We used manipulative field and laboratory-based experiments in which we disrupted microbial communities to determine the role of the host-associated microbiota in host reproductive output, and the effect of both host- and benthic-associated microbiota on the settlement and morphogenesis of non-motile algal propagules (zygotes). If microorganisms are important drivers of host reproduction and settlement, we hypothesized that: (i) disruption to the hosts’ microbiota would negatively affect reproductive output, and (ii) the disruption to the host and/or benthic microbiota would negatively affect settlement of zygotes.

## 2. Methods

### (a) Effect of disruption of the host microbiota on reproductive output

In total, 120 female *H. banksii* of approximately 0.8 ± 0.3 cm in diameter and 8.2± s.e. 1.7cm in length, separated by approximately 1 m, were tagged at Cape Banks Aquatic Reserve, Sydney, Australia (33°59'55.3"S 151°14'53.6"E), during low tide in December 2022. Female individuals can be differentiated from males within the field by examining the reproductive structures as males of this species have orange conceptacles (due to the coloration of the sperm; electronic supplementary material, figure S1). In total, 30 female individuals were assigned to each of four treatments: (i) control, *Hormosira* left undisturbed, (ii) procedural control (AFSW), 100 ml of autoclaved filtered seawater (AFSW) applied once for 4 h, (ii) antibiotics AB2 (penicillin (100 mg/ml), neomycin (100 mg/ml) and rifampicin (50 mg/ml), in 100 ml of AFSW applied once for 4 h, and (iv) antibiotics AB3 (penicillin (100 mg/ml), streptomycin (25 mg/ml), norfloxacin (1 mg/ml), kanamycin (25 mg/ml), neomycin (100 mg/ml) and chloramphenicol (10 mg/ml) in 100 ml of AFSW applied once for 4 h. To ensure the treatments did not affect the surrounding algal individuals, a ring of sponges was placed around each individual while the treatment was applied to absorb any additional liquid. The ring was then removed after 3 h as the tide encroached, and the treatment was rinsed off the algae by the tide. The antibiotic treatments were based on earlier work [58] in which we provided evidence that the antibiotic mixes used in treatments 3 and 4 significantly affected the host microbiota but did not have a discernible direct effect on the host (i.e. a chemical effect on the host), thus in principle allowing for testing of microbially mediated effects on host performance rather than direct effects of the antibiotics on the host [58].

All individuals were collected from the field after 2 weeks, as a previous study showed that the greatest effect of microbial disruption on host function was observed after this time period, which was correlated with microbial composition [58]. Individuals were then transported within 40 min to the laboratory at The University of Sydney, refrigerated for 12 h at 4°C and placed under high light (approximately 500 lux) in individual containers to induce spawning (adapted from [66]; electronic supplementary material, figure S2). Five individuals from each treatment were removed before spawning was induced to destructively sample the bacterial communities (described below). To measure reproductive output, the number of eggs released from each individual was counted before fertilizing the eggs through the introduction of 1 ml of sperm (optical density at 600 nm -OD600) suspended in AFSW into each individual’s tank before leaving them for 30 min to fertilize (as per [66]). Sperm was collected from a common group of 20 males who were kept separate from the experimental individuals, but which were collected at the same time from the same field site and spawned concurrently with the females.

### (b) Effect of disruption of host and benthic microbiota on zygote settlement

To compare the effect of host- versus benthic-associated microbiota on the settlement and morphogenesis of *Hormosira’*s zygotes, 100 sandstone tiles (12 × 12 cm side length, 1 cm thickness) were installed at Cape Banks Aquatic Reserve intertidal, sandstone rocky shore 2 weeks prior to treatment (electronic supplementary material, figure S3). This allowed a natural biofilm to develop [67] on the tiles, while limiting the time available for colonization of macroorganisms which could confound analyses. Tiles were assigned to 1 of 5 treatments within the field: 1−4 were the same treatments as those applied to the host but applied on the tile biofilms (described above), with the addition of 5) tile sterile (autoclaved; tile autoclaved after collection). This last treatment (TS) was to test whether the bacteria within the biofilm had to be alive to induce settlement. Tiles were then collected after treatment and transported to the aquarium at The University of Sydney where they were placed within individual tanks (electronic supplementary material, figure S2). To ensure no zygotes of *Hormosira* (visible under magnification) had already been attached to the tiles in the field, tiles were checked on collection using a hand lens (20 × Danoplus).

Zygotes, released and fertilized by the macroalgal individuals used in the host experiment described above, were introduced to the tiles in an experimental design in which all host and tile treatments were crossed (20 treatment combinations; *n* = 5 tiles and females per treatment combination). To control for potential density-dependent effects, zygotes were diluted to the lowest concentration obtained (200 ± s.e. 23 zygotes per 50 ml) from each individual (*n* = 5 per treatment). Zygotes were allowed to attach for 4 h, the duration of average emersion during low tides at Cape Banks. Then, each plate was flushed using 50 ml of AFSW to remove any unattached zygotes and the remaining number of settled zygotes were counted using a dissecting microscope (Leica MZ12).

### (c) Bacterial sampling

To collect surface-associated microbiota for characterization, the surface of each alga and tile was swabbed for 30 s with a sterile cotton swab [59,68]. Swab controls (treated similarly to those for swabbing surfaces, except no surfaces were swabbed) were also collected. Swabs were immediately placed into sterile cryogenic tubes and placed in liquid nitrogen and then stored at −80°C until DNA extraction.

### (d) DNA extraction and sequencing

Microbial DNA was extracted from each swab sample in a randomized order to avoid introducing any bias due to order and time of processing, using a Powersoil DNA Isolation kit (Qiagen) following the manufacturer’s protocol. DNA extracts were quantified using spectrophotometry (Nanodrop 1000) and stored at −20°C until sequencing.

The extracted DNA samples were amplified with polymerase chain reaction (PCR) using the 16S ribosomal DNA (rDNA) gene primers 341 (F) (5’- CCTACGGGNGGCWGCAG-3’) and 805(R)—(5’-GACTACHVGGGTATCTAATCC-‘3), containing the V3–V4 regions of the bacterial and archaeal 16S rRNA gene [69]. The PCR conditions involved a pre-heating step to 95°C for 3  min followed by 35 cycles of 95°C for 15  s, 55°C for 1  min and 73°C for 30  s. Both positive (with known DNA sequences) and negative controls (nuclease-free water, control swabs) were used. The negative controls did not amplify DNA, suggesting no contamination of swabs or during extraction and amplification. Agarose gel electrophoresis and Nanodrop 1000 were used to ensure the quantity and quality of the amplicons before they were sent for sequencing via the Illumina MiSeq 2000 platform at the Ramaciotti Centre for Genomics (University of New South Wales, Sydney). While previous work has shown potentially spurious sequences arising from sequencing [70], the inclusion of swab controls allowed us to remove taxa arising from both sequencing errors and contamination in processing. Furthermore, our transformation of the data using quantitative PCR (qPCR, described below) overcomes another limitation of 16S rRNA gene sequencing as it provides a clearer representation of the true abundances of these taxa across and within samples.

### (e) Bioinformatics

Raw sequences were received from the sequencing centre as demultiplexed pair-ended sequences per sample. UNOISE was used to remove chimeras and produce amplicon sequence variants (ASVs) at a unique sequence level (0% distance) [71]. DADA2 was used to map the original reads back to ASVs, generating a table of 11 847 ASVs [72]. ASV sequences were searched with BlastN against the SILVA SSU Ref NR99 database for taxonomic classification and to remove chloroplasts. The Genome Taxonomy Database (GTDB) was then used for taxonomic assignment. Singletons and low abundance taxa (<0.01% of reads) were removed from the dataset prior to statistical analyses, resulting in 8075 ASVs.

### (f) Estimation of absolute bacterial abundance

Total abundance of the 16S rRNA gene was quantified for each sample by qPCR using the primers 341F/805R [73]. Gene amplification and analysis were performed using the QuantStudio 3 thermocycler (Thermo Fisher with PrimeTime® Gene Expression Master Mix, Integrated DNA Technologies) and associated software. The reaction conditions for amplification of DNA were 50°C for 2 min, 95°C for 10 min and 40 cycles of 95°C for 15 s and 55°C for 1 min. The final gene copy number per sample was corrected for the total extraction volume, the surface area, the dilution factor and DNA yield per sample (see [74] for further details) and were used to estimate absolute abundances of ASVs.

### (g) Statistical analyses

**Figure 1.**
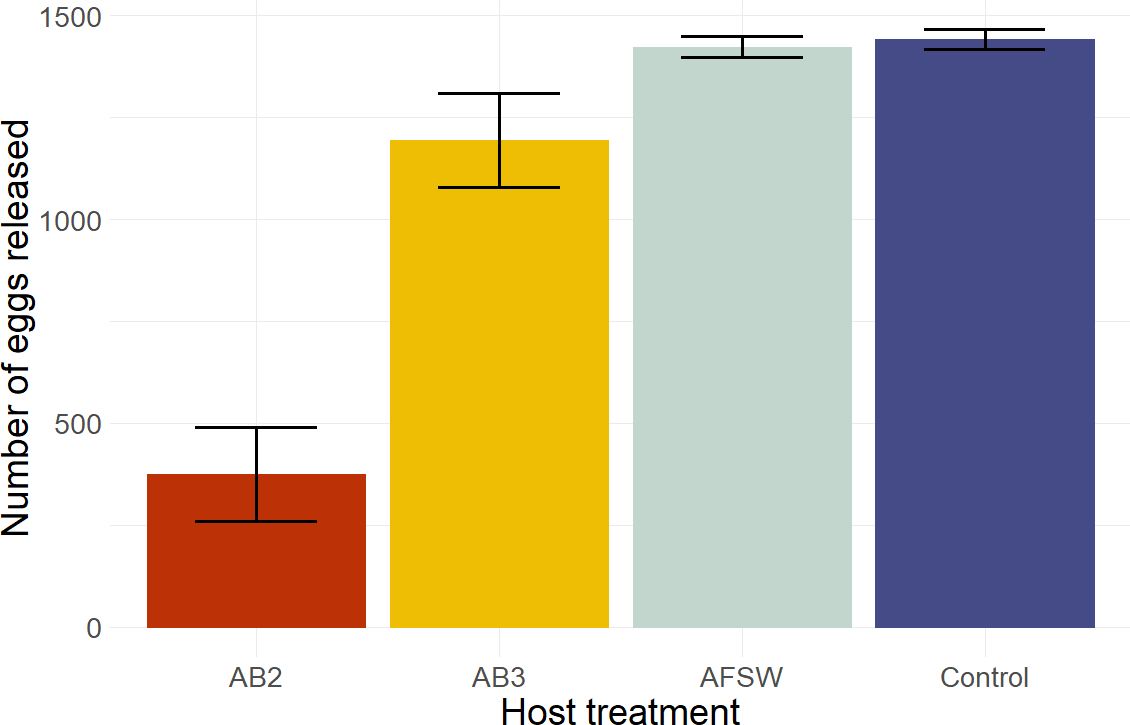
Average number of eggs released by *Hormosira* from each treatment (means ± s.e.; *n* = 25).

To test for the effects of manipulations of microbiota on host reproductive output, we used a one-factor ANOVA (fixed, 4 levels) and to test for the effect of host and tile microbial disruption on zygote settlement we used a two-factor ANOVA (host and tile factors, fixed, crossed, 4 and 5 levels, respectively) using the GAD package in R (v.4.0.3). The normality of the data was assessed using a Shapiro–Wilks test with a Levene’s test being used to test homoscedasticity. *Post hoc* contrasts (emmeans R package) were used to examine significant main effects or interactions, as appropriate. Further to this, we calculated the multiplicative effect of host and tile treatment by predicting the total number of eggs (assuming no density-based effects) that would have settled based on the percentage observed along with the total released by each individual (settled/total released – figure 1).

To account for uneven sequencing depth in bacterial community data among samples, data were normalized using qPCR reads of the 16S rRNA gene, V3−4 regions [74]. To determine differences in the structure of the associated bacterial assemblages, the normalized ASV data were analysed using permutational multivariate analyses of variance [75] based on Bray–Curtis dissimilarities between sample pairs, calculated on square-root transformed absolute (qPCR normalized) abundances of ASVs in the R vegan package [76]. Two analyses were done, one for host-associated bacterial communities (host treatment as a fixed factor with 4 levels) and one for tile-associated bacterial communities (tile treatment as a fixed factor with 5 levels). For significant main effects, *post hoc* contrasts were performed using the pairwise.adonis.2 function in the *pairwise.adonis* package [77].

Generalized linear latent variable models (GLLVMs) were used to visualize the multivariate models using ordination plots (see [78]). GLLVMs were used as they provide a model-based ordination method rather than unconstrained algorithmic approaches (e.g. nMDS). The GLLVMs used the same model structure as the multivariate analyses of variance with a negative binomial distribution, which was selected after comparing model residuals with other distributions. To determine which bacterial taxa differed among treatments, we used multivariate generalized linear models (GLMs; host or tile factors as above) using the R package ‘mvabund’ [79] assuming a negative-binomial distribution to account for over-dispersion of sequence counts.

## 3. Results

### (a) Effect of treatments on host- and tile-associated microbiota

**Figure 2.**
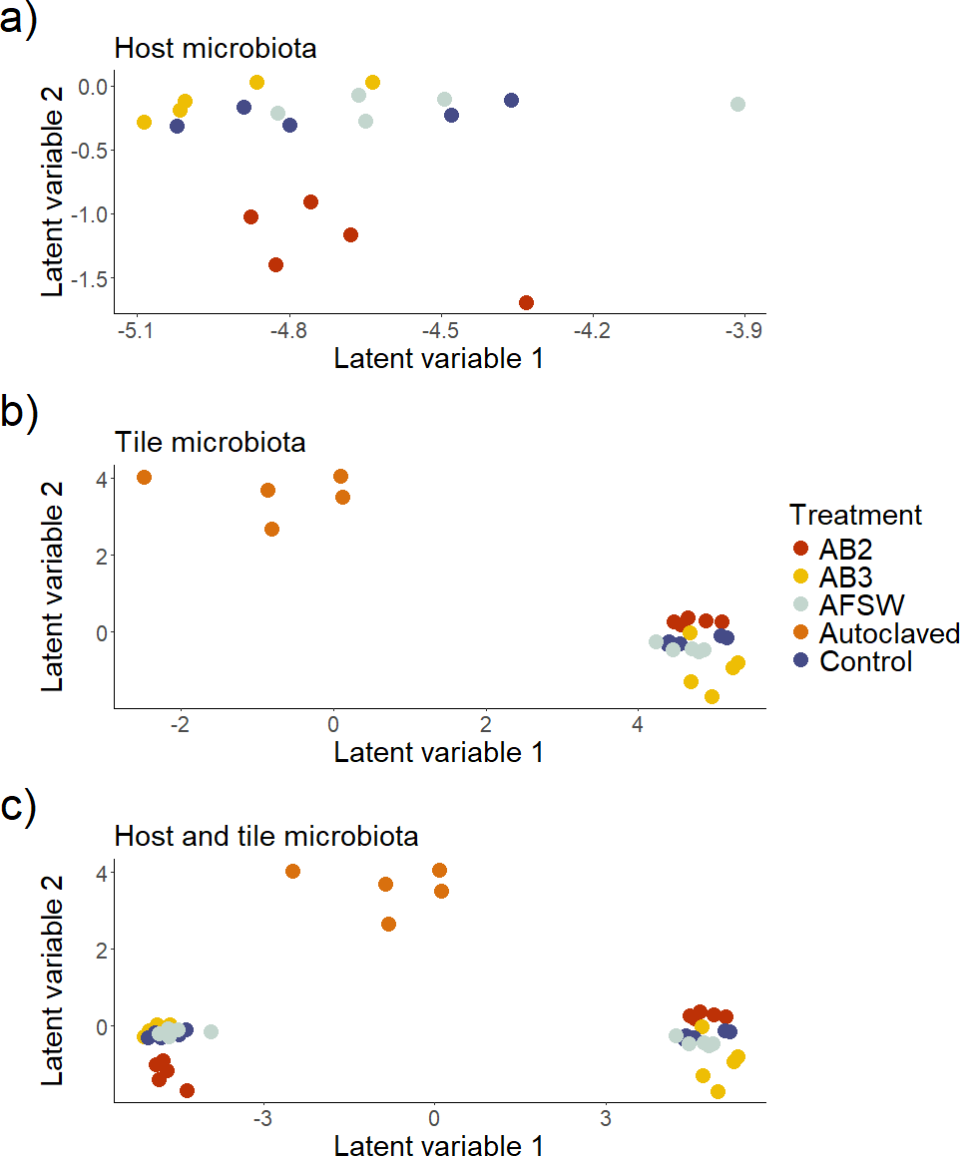
Ordinations based on GLLVM models [78] of bacterial community structure on hosts (a), tiles (b) or their combination ((c), including autoclaved treatment) after 2 weeks. Host (circles) and tile (triangles) treatments: antibiotics AB2 (red symbols), AB3 (yellow symbols), procedural control (AFSW; grey symbols), control (blue symbols) and autoclaved tiles (orange symbols; *n* = 5).

The bacterial community structure on *Hormosira* individuals differed between treatments AB2 and AB3, which in turn, differed from controls after 2 weeks (pseudo-F_3,17_ = 6.74, *p* < 0.01, electronic supplementary material, table S1; figure 2a,c). Similarly, the structure of bacterial communities on autoclaved, AB2 and AB3 tiles differed from each other and these communities, in turn, differed from those on control tiles after 2 weeks (pseudo-F_4,21_ = 9.60, *p* < 0.01, electronic supplementary material, table S2; figure 2b,c).

**Figure 3.**
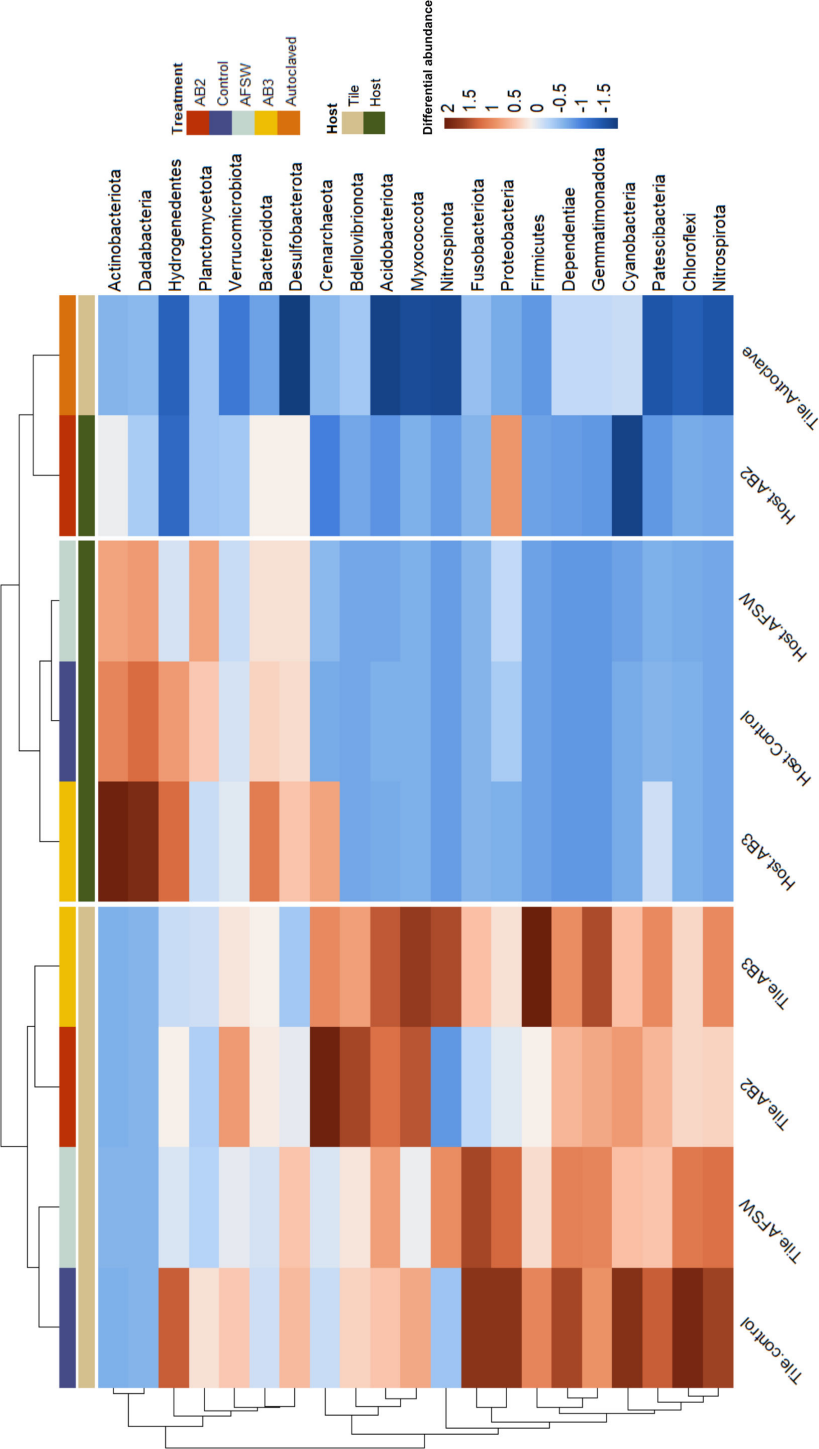
Heatmap showing the significant log-fold changes in phyla abundance between host type (tile or alga) and treatment across samples. Differences are based on the average Euclidean difference in the abundance of each taxon from each sample/treatment combination (*n* = 5). The tree at the top of the plot indicates clusters of treatments based on their Euclidean distances from one another. The tree to the left of the plot shows the hierarchical clustering of the different taxa based on their Euclidean distances from one another.

GLM analyses identified 246 ASVs associated with the host (approximately 2.0% of a total of 12 140 ASVs, electronic supplementary material, table S3) whose abundances differed significantly among treatments. Of those, we found a significant negative effect of treatment on 13 ASVs within the following taxa *Vibrio, Alteromonas, Marinomonas, Geminocystis, Phormidesmiaceae, Pleurocapsa* with decreases in abundances ranging from 13% to 62% (figure 3; electronic supplementary material, table S3 and figure S4) in the AB2 treatment compared with all other treatments.

GLM analyses identified 189 ASVs associated with the tiles (approximately 1.5% of a total of 12 140 ASVs, figure 3; electronic supplementary material, table S4) whose abundances differed significantly among treatments. Of those, we found a significant negative effect of treatment on 17 ASVs belonging to the taxa *Vibrio, Alteromonas, Marinomonas, Pleurocapsa* and *Prochloraceae*. The abundance of these ASVs was significantly lower (16%–48%; figure 3; electronic supplementary material, table S4 and figure S4) in the AB2 treatment compared with other treatments, and all taxa were >85% lower in the autoclaved treatment than in other treatments.

### (b) Effect of microbial disruption on reproductive output

Disruption of the host microbiota with antibiotic treatment AB2 resulted in a significantly lower number of eggs released than hosts treated with AB3 or controls, which did not differ from each other (ANOVA, F_3,96_ = 812.7, *p* < 0.001, figure 1, electronic supplementary material, table S5).

### (c) Effect of microbial disruption on settlement of zygotes

**Figure 4.**
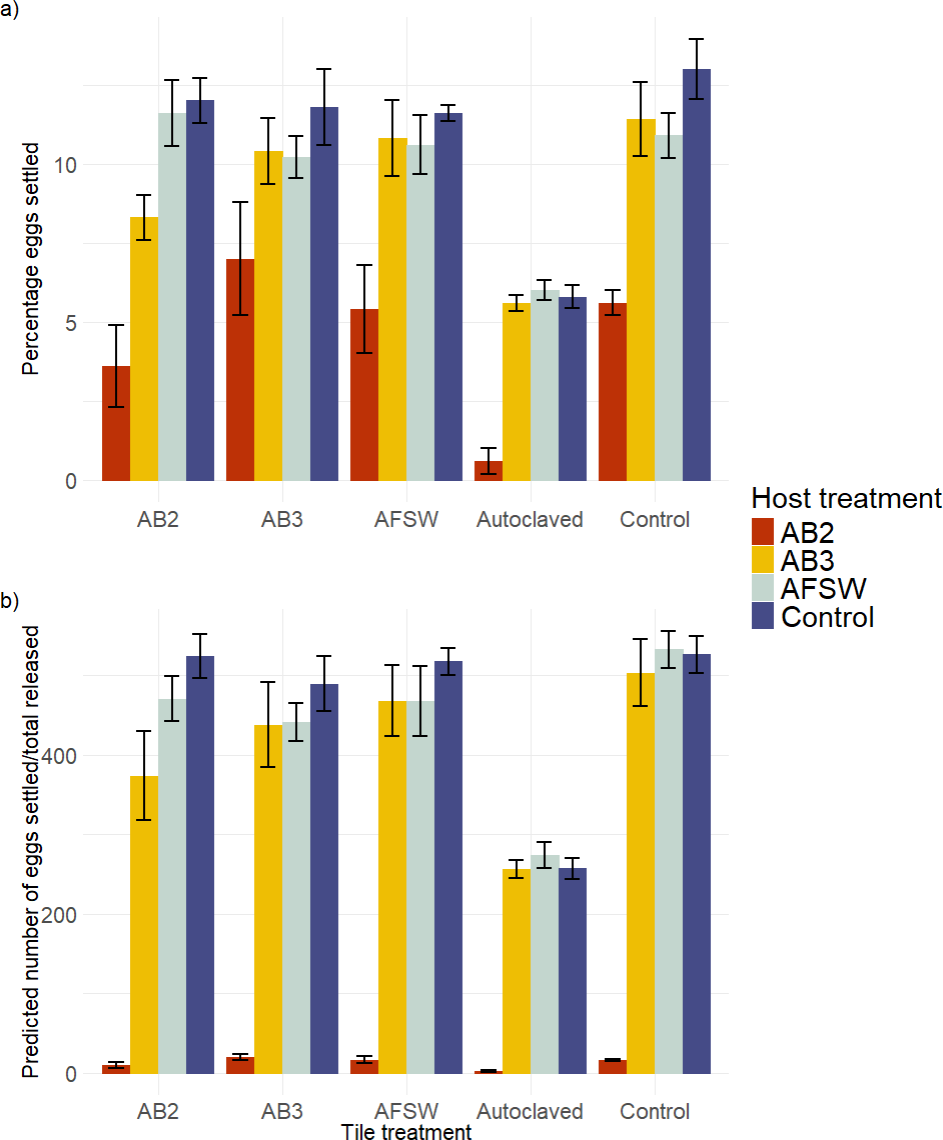
(a) Percentage of eggs that settled (out of 200 added) onto each tile after 4 h (means ± s.e., *n* = 5 tiles per host and tile treatment combination). Host treatments: antibiotics AB2 (red bars), AB3 (yellow bars), procedural control (AFSW; grey bars), control (blue bars). (b) Predicted number of eggs was calculated by taking the percentage settled out of the total number of eggs released.

Disruption to the host- and tile-associated microbiota caused significant, but complex and interactive, effects on the settlement of *Hormosira’s* zygotes (ANOVA, F_12,80_ = 0.771, *p* < 0.001, electronic supplementary material, table S6, figure 4). *Post hoc* contrasts showed hosts treated with AB2 had significantly lower levels of settlement than hosts treated with AB3 or controls and this was consistent across all tile treatments (electronic supplementary material, table S2). Settlement levels for hosts treated with AB3 generally did not differ from controls except when combined with tiles treated with AB2, for which settlement levels of hosts treated with AB3 were lower than controls (but higher than hosts treated with AB2; electronic supplementary material, table S6). When AB2 was applied to the host in combination with autoclaving the tiles, this treatment combination had the lowest settlement level of all treatments (0.64% ± 0.43%, *n* = 5, figure 4).

Furthermore, *post hoc* tests showed that all tiles where the microbiota was disrupted by autoclaving had significantly lower levels of settlement (by approximately 7%) than those that were not autoclaved, which was generally consistent across all host treatments (electronic supplementary material, table S6). Tiles treated with AB2 did not affect the settlement of zygotes unless this treatment was combined with host disruption (AB2 or AB3).

## 4. Discussion

Evidence from a diverse taxa show that host-associated microbiota are fundamentally important for host function and performance [80–82]. Here, we show that host-associated microbiota are critical for a macroalgal host at several stages of their life cycle. We show that disruption to host-associated microbiota affected host reproductive output. Furthermore, microbiota associated with both the adult host and the benthic substratum had a strong interactive effect on zygote settlement and morphogenesis. Both effects likely have longer-term consequences for these macroalgae, and the persistence of the ecosystems they support. Measures of host function within macroalgae have typically used physiological measures such as photosynthetic yield [58,60,68], nutrient acquisition [83,84] or respiration [85,86]. However, such physiological measures, which typically represent a small snapshot of an organisms’ life history, may not accurately represent long-term performance of the host or its offspring, i.e. fitness. Here, measures with tangible links to organisms’ fitness were used to help understand how microbiota can influence elements of host fitness at different stages of a host’s life.

An unexpected finding, based on the previous literature, was the extent to which host-associated microbiota influenced offspring settlement versus the importance of tile microbiota on settlement (see [29] and [40]). While contrasts showed that disruption to the tiles caused a significant effect on the settlement of zygotes across host treatments, the strongest negative effect was the combination of disruption of the host with antibiotics when combined with tile disruption (autoclaved); however, this effect was antibiotic specific. Zygotes released from adults whose surface-associated microbiota were disrupted with AB2 had significantly lower levels of settlement than controls across tile treatments, whereas hosts treated with AB3 did not influence settlement unless the tile was also disrupted. As the eggs are released, they may acquire microbiota from the host through contact with the external follicles surrounding the sexual conceptacles which may influence subsequent settlement or morphogenesis.

It is possible that disruption to the host microbiota may potentially affect the transfer of beneficial microbes from adults to their gametes and eventually zygotes through vertical transmission. Vertical transmission of the adult microbiota has recently been shown for *Hormosira* [87]. Vertically transmitted microbes have been shown to be important drivers of early life-stage survival and morphogenesis of hosts ranging from corals to sponges [88,89]. Furthermore, vertically transmitted microbes have been suggested to cause downstream effects on offspring fitness within a few ‘model’ host species [90,91]. To explore the mechanisms underlying this observation, further work could examine the microbiota associated with the host, gametes and zygotes, in both disrupted and undisrupted individuals (e.g. [92]).

The microbial disruption treatments used here substantially changed the host and tile microbiota, particularly for the AB2 treatment, which significantly changed the abundance of 104 host-associated bacterial ASVs. Contrastingly, while AB3 caused significant changes in the bacterial community structure, and the abundance of 38 bacterial ASVs, it did not cause a significant effect on the reproductive output or settlement of *Hormosira*. Most of the bacteria significantly reduced by the AB2 treatment belonged to the phyla Cyanobacteria, while the majority of those whose abundances changed within AB3 were from the class Gamaproteobacteria. Cyanobacteria are important microbial consorts in a number of marine organisms, ranging from sponges [93,94] to macroalgae [84], and are often important components of the nitrogen-acquisition capabilities of their hosts [61,95]. Indeed, within the class Cyanophyceae, we found a number of known symbionts of other marine holobionts capable of nitrogen fixation and belonged to the families Acaryochloridaceae and Synechococcaceae (electronic supplementary material, figure S4 [61,89,95]). The acquisition of nitrogen for these algal holobionts is especially important to understand, particularly in intertidal systems where nitrogen is often limited [96–98]. As nitrogen is a key nutrient required for growth and development [83,89] the potential loss of nitrogen sources from the removal of Cyanophyceae may reduce nitrogen availability for the host. This could, in turn, lead to a lower investment of nutrients into reproductive output, as is often seen within terrestrial plants in nutrient-scarce environments [99–101]. Future work could incorporate stable isotope probing to determine the relative role of microbial taxa in the acquisition of nitrogen within this species [102].

Disruption to the microbiota on the settlement tiles also had a significant effect on the settlement of *Hormosira*’s zygotes across host treatments. Autoclaving the tiles caused the greatest disruption to the bacterial community structure and the strongest reduction in settlement of *Hormosira*, irrespective of host treatment, though some settlement still occurred. Autoclaving is very successful in sterilizing surfaces [103,104], indicating that live bacterial cells appear to be important cues for the settlement of this species. Settlement of marine invertebrates has been strongly correlated with the presence and abundance of the benthic biofilm, as well as specific microbial taxa such as Gamaproteobacteria (e.g. *Vibrio* sp. and *Alteromonas* sp.) and Cyanobacteria (e.g. *Pleuocaspa* sp. and *Geminocycstis* sp.) [32,105]. This contrasts with evidence from other hosts such as bryozoans [106] and some cnidarians [38,107] which respond to broader, non-specific microbial or environmental cues for settlement and morphogenesis. These broader microbial cues have often been correlated with the age of the biofilm which has been shown to be an important factor in the settlement and morphogenesis of marine larvae [9,39,108]. However, as all benthic biofilms were the same age (2 weeks), the age of these biofilms cannot explain the observed differences in zygote settlement. Autoclaved tiles had the lowest bacterial densities and rate of successful settlement, which were related. While this pattern has been observed in other invertebrates [14,29], more work is required to determine the mechanisms driving the observed effects.

### (a) Synthesis

Recruitment (propagule settlement, morphogenesis and survival) can explain much of the spatial or temporal variation in population dynamics in marine organisms [109–111]. Understanding the drivers of recruitment is an important step in determining lifetime fitness and population persistence [112]. While there is a large body of literature showing recruitment patterns for multiple seaweed species [113–115], the role of microbiota in mediating these key life-history processes for seaweeds is poorly known (but see [47]). Here, we show that microbiota associated with both the host and the benthos have important roles in key stages of a host's life cycle: reproductive output and settlement. Future work should examine the molecular mechanisms underpinning these observations and incorporate sampling to test for vertical transmission of microbes. Understanding these factors which potentially control habitat-forming hosts’ fitness across their life cycles is important for how we consider the ecology of these habitat-forming organisms in the Anthroprocene.

## Data Availability

All data; amplicon sequence data, host responses and the code to reproduce the figures within this paper are available on Dryad [116]. Supplementary material is available online [117].
